# Several sources of error in estimation of left ventricular mass with M-mode echocardiography in elderly subjects

**DOI:** 10.3109/03009734.2011.596586

**Published:** 2011-10-29

**Authors:** Charlotte Ebeling Barbier, Lars Johansson, Lars Lind, Håkan Ahlström, Tomas Bjerner

**Affiliations:** ^1^Department of Radiology, Uppsala University Hospital, Sweden; ^2^AstraZeneca, Gothenburg, Sweden; ^3^Department of Medicine, Uppsala University Hospital, Sweden

**Keywords:** Left ventricular mass, magnetic resonance imaging, M-mode echocardiography

## Abstract

**Introduction:**

M-mode echocardiography estimates of the left ventricular mass (LVM) were greater than magnetic resonance imaging (MRI) estimates. There are substantial differences between the methods both in the means of measuring and the calculation formula. The aim of this study was to investigate whether any difference in estimates of LVM between M-mode echocardiography and MRI is due to the means of measuring or to the calculation formula, using MRI as the gold standard.

**Material and methods:**

M-mode echocardiography and MRI were performed on 229 randomly selected 70-year-old community-living subjects. LVM was calculated from echocardiography (LVM_echo_) and from MRI (LVM_MRI_) measurements using standard techniques. Additionally LVM was calculated with the echocardiography formula from echo-mimicking measurements made on MR images (LVM_MRI/ASE_).

**Results:**

There were significant differences between all three LVM estimates in women, in men, and in the entire population. Echocardiography estimated LVM to be larger than did MRI, and the LVM_MRI/ASE_ estimate was larger than the LVM_MRI_. The difference between LVM_MRI_ and LVM_MRI/ASE_ was larger than the difference between LVM_echo_ and LVM_MRI/ASE_. There was a low correlation between LVM_echo_ and LVM_MRI_ (*R^2^* = 0.46) as well as between LVM_MRI/ASE_ and LVM_MRI_ (*R^2^* = 0.65).

**Conclusion:**

The means of measuring and the calculation formula both independently add to the error in LVM estimation with M-mode echocardiography. The error of the calculation formula seems to be greater than the error of the means of measuring in a population of community-living elderly men and women.

## Introduction

Estimation of the left ventricular mass (LVM) is an important diagnostic and prognostic tool in patients suffering from various forms of heart disease. Estimations can be made with different modalities. M-mode echocardiography is widely used in clinical and scientific practice, despite its lacking accuracy and reproducibility (1–3). The availability of magnetic resonance imaging (MRI) is more limited. MRI is, however, very accurate and reproducible (4–7) and frequently considered the gold standard for LVM estimation (8–10).

M-mode echocardiography estimates of LVM are greater than MRIestimates (11–13) which can be explained by several factors. There are substantial differences between the methods both in the means of measuring and in the calculation formula.

Current notions of increased LVM being a predictor of increased morbidity and mortality are based on epidemiologic studies using M-mode echocardiography to determine LVM (14–16). Investigation of the error in this technique is thus of interest.

The first aim of the present study was to compare LVM estimated with echocardiography to LVM estimated with MRI, in the same 70-year-old subjects, using MRI as the gold standard. The second aim was to investigate whether any detected difference in LVM estimates is due to the means of measuring or to the calculation formula.

## Material and methods

### Study population

After obtaining approval from the Ethical Committee and written informed consent, the Prospective Investigation of the Vasculature in Uppsala Seniors (PIVUS) study (17) conducted studies including echocardiography on 1,016 randomly selected subjects who were recruited at 70 years of age (participation rate 50.1%).

Magnetic resonance imaging (MRI) was performed on 293 consecutively invited subjects from the original cohort. A subsample of 229 subjects (113 women, 116 men), who had assessable MR images and echocardiography results, constituted the population of the present study. The MRI was performed within 3–22 months (mean 16 months) of the echocardiography.

The basic characteristics among these subjects did not differ from those in the entire PIVUS population(Table I) (17).

**Table I.** Basic characteristics of subjects (mean ± standard deviation).

	Total PIVUS sample	This sample
*n*	1016	229
Females (%)	50.2	49.3
Height (cm)	169 ± 9.1	169 ± 9.3
Weight (kg)	77 ± 14	76 ± 13
Waist circumference (cm)	91 ± 12	90 ± 9.8
Body mass index (kg/m^2^)	27.0 ± 4.3	26.5 ± 3.6
Systolic blood pressure (mmHg)	150 ± 23	147 ± 19
Diastolic blood pressure (mmHg)	79 ± 10	78 ± 9.6
Heart rate (beats/min)	62 ± 8.7	61 ± 8.7

### Echocardiography

Echocardiography was performed by one observer using an Acuson XP124 cardiac ultrasound unit (Acuson, California, USA) with a 2.5 MHz transducer.

#### LVM_echo_

M-mode echocardiography was performed from the parasternal short-axis view, using a leading-edge-to-leading-edge technique. The cursor was placed apically to the mitral valve so that no mitral valve movement was seen in the M-mode recordings. Harmonic imaging was not available on the equipment used.

Measurements included the interventricular septum thickness (IVS), the posterior wall thickness (PW), and the left ventricular inner diameter in end-diastole (LVEDD). LVM was derived from the following formula (equation 1), validated by Devereux et al. using the American Society of Echocardiography (ASE) convention (18):

(Eq.1) $$\mathrm{LV}M_{\mathrm{echo}}\;=\;0.8\;(1.04\;({\left[\mathrm{LVEDD}+\mathrm{PW}+\mathrm{IVS}\right]}^{3}))\;+\;0.6g$$

### MR image acquisition

MRI was performed on a 1.5 teslaMRI system (Gyroscan Intera; Philips Medical Systems, Best, The Netherlands) with a 25 mT/m gradient system, using the standard SENSE-cardiac coil in the supine position and retrospectively gated vector-ECG for cardiac triggering.

A steady-state free precession (SSFP) cine sequence was used covering the left ventricular myocardium in 8 mm thick short-axis slices with a 2.5 mm slice gap. The number of slices was adjusted to cover the heart from the apex to the atria. Two slices were acquired per breath-hold (14 s) with an acquired in-plane resolution of 2.27 × 1.81 mm (reconstructed to 1.56 × 1.56 mm). The following parameters were used: TR = shortest (∼3.6 ms), TE = shortest (∼1.8 ms), flip angle = 70°, bandwidth = 723.8 Hz/pixel, 18 phases/cardiac cycle, field of view (FOV) = 400 mm, matrix = 256, parallel imaging (SENSE) factor = 2, and k-lines segments (TFE-factor) = 19.

### MR image analysis

Image analysis was performed by one observer using commercially available analysis software (ViewForum; Philips Medical Systems, Best, The Netherlands). The observer was blinded to the echocardiography results. The first phase following the ECG Rwave was defined as end-diastole.

#### LVM_MRI_

Border definition of the epicardial contour was accomplished by manual tracing using a mouse or a pen tablet (Wacom, Washinomia Industrial Park, Saitama, Japan). The endocardial contour was generated with computer assistance, where a manually drawn contour is automatically adapted to the underlying image. The papillary muscles were included in the endocardial contour when attached to the ventricular wall. Papillary muscles not attached to the wall were included in the LV blood volume. The end-diastolic LVM was calculated by the software using Simpson's rule approximation and assuming a myocardial density of 1.05 g/mL (LVM_MRI_).

#### LVM_MRI/ASE_

**Figure 1.**
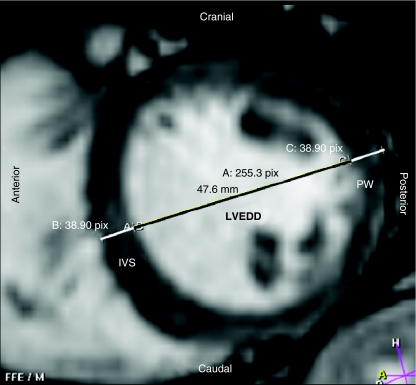
Measurements on short-axis magnetic resonance images were placed to correspond with measurements made on M-mode echocardiography and were used to calculate the left ventricular mass with the formula commonly used in echocardiography (LVM_MRI/ASE_). The end-diastolic inner left ventricular diameter (LVEDD) was measured from the middle of the inner aspect of the septal wall to the inner aspect of the posterior wall, and the interventricular septal thickness (IVS) and the posterior wall thickness (PW) were measured at the same level. To correspond with a parasternal short-axis M-mode view the image has to be turned 90 degrees clockwise.

The maximal inner LV diameter was measured in end-diastole (LVEDD)from the middle of the inner aspect of the septal wall to the inner aspect of the posterior wall (Figure 1). To obtain the maximal diameter, measurements were made in the most cranial diastolic slice where the papillary muscles appeared detached from the myocardial wall, in order to correspond to the level where the LV diameter was measured at echocardiography. The diameters in the adjacent slices above and below were also measured, and the top value was selected.

The IVS and PW were measured on MR images at the same level as the LVEDD (Figure 1). LVM_MRI/ASE_ was calculated from these measurements using the same formula as for echocardiography (see above) (18).

### Statistical analysis

StatView 5.0.1 (SAS Institute, Cary, North Carolina, USA) was used for statistical analyses. A paired *t*test was used to estimate differences between the LVM measurements. A regression analysis was performed to estimate how LVM_echo_ and LVM_MRI/ASE_ correlated to LVM_MRI_. The significance level was set at 0.05 in all analyses.

## Results

LVM_MRI_, LVM_echo_, and LVM_MRI/ASE_ measurements are presented in Table II. There were significant differences (*p* < 0.0001) between all three LVM estimates in women, men, and in the entire population (Table II).

**Table II.** Left ventricular mass (mean ± standard deviation) in women, men, and the entire population calculated from measurements on MR images and echocardiography.

	LVM_MRI_	Difference (LVM_MRI/ASE_–LVM_MRI_)	LVM_MRI/ASE_	Difference (LVM_echo_–LVM_MRI/ASE_)	LVM_echo_	Difference (LVM_echo_–LVM_MRI_)
Women (*n* = 113)	91 ± 20 g	35 ± 4^a^	126 ± 35 g	25 ± 7^a^	154 ± 48 g	60 ± 6^a^
Men (*n* = 116)	136 ± 30 g	39 ± 6^a^	175 ± 50 g	27 ± 10^a^	200 ± 59 g	64 ± 8^a^
Total (*n* = 229)	114 ± 34 g	37 ± 3^a^	151 ± 50 g	26 ± 6^a^	177 ± 59 g	62 ± 5^a^

^a^*P* < 0.0001. LVM = left ventricular mass; LVM_echo_ = LVM estimated with M-mode echocardiography using standard techniques; LVM_MRI_ = LVM estimated with magnetic resonance imaging using standard techniques; LVM_MRI/ASE_ = LVM calculated with the echocardiography formula from echo-mimicking measurements made on MR images.

LVM_echo_ measurements were the greatest, LVM_MRI/ASE_ were intermediate, and LVM_MRI_ were the smallest (Table II). The difference between LVM_MRI_ and LVM_MRI/ASE_ was larger than the difference between LVM_echo_ and LVM_MRI/ASE_ (Table II).

**Figure 2.**
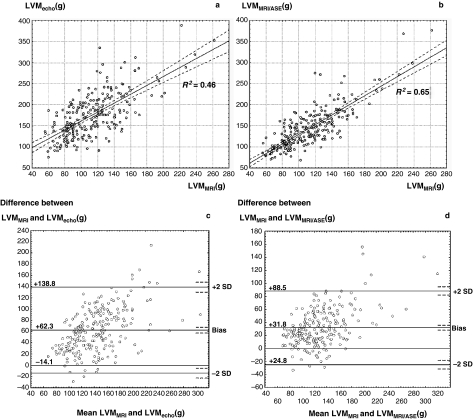
Regression analysis of left ventricular mass (LVM) estimates made with M-mode echocardiography (LVM_echo_) and with magnetic resonance imaging (LVM_MRI_) using standard techniques (A), and calculated with the echocardiography formula from echo-mimicking measurements made on MR images (LVM_MRI/ASE_)(B). Coefficients of determination are displayed in the diagrams. Bland–Altman plots displaying the agreement between the measurements of LVM_MRI_ and LVM_echo_ (C), and between the measurements of LVM_MRI_ and LVM_MRI/ASE_ (D).

LVM_echo_ correlated to LVM_MRI_ with an *R^2^* of 0.46 (regression coefficient = 1.07), and LVM_MRI/ASE_ correlated to LVM_MRI_ with an *R^2^* of 0.65 (regression coefficient = 1.15) (Figure 2).

## Discussion

The observation that there were significant differences between all three LVM estimates implies that both the means of measuring and the calculation formula, independently of each other, add to the error in M-mode echocardiography. This notion is endorsed by the low correlations that were observed between LVM_echo_ and LVM_MRI_ (*R^2^* = 0.46) as well as between LVM_MRI/ASE_ and LVM_MRI_ (*R^2^* = 0.65). The observation that the difference between LVM_MRI_ and LVM_MRI/ASE_ was larger than the difference between LVM_echo_ and LVM_MRI/ASE_ implies that the error of the calculation formula is greater than the error caused by the means of measuring.

LVM_MRI_ (13) and LVM_echo_ (19) measurements were within the range of previously observed dimensions in corresponding populations, and the observation that echocardiography estimated LVM to be greater than did MRI is also consistent with observations made by others (11–13,20).These observations probably reflect the general inadequacy of M-mode echocardiography LVM estimates to be expected in a population of community-living elderly men and women.

The parameter LVM_MRI/ASE_ was calculated using the formula commonly used in echocardiography but from MRI measurements performed in order to correspond with measurements made on M-mode echocardiography. This parameter was created to separate the measurement variability from the potential inadequacy of the calculation formula.

LVM has been calculated from MRI measurements using an echocardiography formula before (21). That study was, however, performed on hypertensive patients, and a spin-echo MRI sequence was used; MRI measurements were made in end-systole, whereas echocardiography was performed in end-diastole, and older calculation formulas were used (21). The present study was performed on community-living subjects and current standard techniques were used, i.e. an SSFP MRI sequence, all measurements were made in end-diastole, and an improved calculation formula was used (18). Despite these differences, the results were similar to those of the present study and were considered to be largely the result of the geometrical assumptions (21).

It is well known that, using M-mode echocardiography, each step in LVM estimation is a potential source of variability (22). There is an interobserver variability in the viewing plane, the timing of measurements in the cardiac cycle, and the exact placement of the measurements. This in turn may be influenced by the interindividual variability between the investigated subjects in age and body constitution (1), as well as the size, shape, and orientation of the heart itself. The measurements are only made at one level and in one dimension, and the calculation formula is based upon geometric assumptions about the structure of the left ventricle (18,22). These assumptions are based on the cube form (18,23). They cannot apply equally to all individuals and may not be valid at all in hypertrophic (3) or distorted (22) ventricles.

LVM estimated with M-mode echocardiography is, despite the drawbacks of this technique, an important predictor of cardiovascular morbidity and mortality (14–16). The error, which is known to increase in hypertrophic (3) and distorted (22) ventricles, might be enhanced by other cardiac abnormalities. The estimate would thus not solely reflect the LVM but includes aspects of abnormal heart shape, which might enhance the correlation to morbidity and mortality. Though coarse, M-mode echocardiography may be an adequate method to identify subjects at risk. However, to find the appropriate prevention or treatment for each individual a more accurate method, that can discriminate between generally increased LVM and heart shape abnormalities, is required.

When LVM is estimated using MRI, all images depicting the left ventricle are segmented, which is bound to be more exact and less susceptible to interobserver variability.

Recent development of the real-time three-dimensional (3D) echocardiography technique allows a more accurate estimation of LVM (24–26) where endo- and epicardial borders are traced in a similar manner to that used in MRI, and the same formula is used to calculate the LVM (Simpson's rule) (25).LVM estimated with this technique correlates well with MRI estimates (25).

M-mode echocardiography has been used to estimate LVM for several decades (23), and it is unlikely that any further improvement of the technique is possible. Measurement in accuracies are unavoidable (1), and a formula based upon geometrical assumptions made from a few one-dimensional measurements is bound to be in appropriate to a various degree. It has even been argued that the potential error in M-mode echocardiography LVM estimation is so large that this technique cannot be recommended either at a single time point or for serial studies in small populations (20). The reason that M-mode echocardiography is still widely used is probably that it is a cheap, easily accessible, and well established method. From an epidemiologic point of view, this makes it a useful method to identify subjects with an increased cardiovascular risk.

The current gold standard technique MRI (8–10), however, has the disadvantages of being expensive, it has a limited availability, and it is more complicated to perform. Thus, it is not likely that MRI will replace echocardiography completely in clinical practice. Furthermore LVM_MRI_ has not yet proved superior to LVM_echo_ in predicting cardiovascular events. Three-dimensional echocardiography could provide the happy medium, but this remains to be verified in prospective studies.

The present study was limited by the fact that only 70-year-old Caucasians were studied, entailing that the results may not apply to other ethnic or age-groups. The rather long time between the echocardiography and the MRI examination should not have influenced the results, since any rapid LVM change is unlikely in this community-based population sample.

In conclusion, both the means of measuring and the calculation formula add to the error in LVM estimation with M-mode echocardiography.The error of the calculation formula seems to be greater than the error of the means of measuring in a population of community-living elderly men and women.
